# Sexually antagonistic selection on genetic variation underlying both male and female same-sex sexual behavior

**DOI:** 10.1186/s12862-016-0658-4

**Published:** 2016-05-13

**Authors:** David Berger, Tao You, Maravillas R. Minano, Karl Grieshop, Martin I. Lind, Göran Arnqvist, Alexei A. Maklakov

**Affiliations:** Animal Ecology, Department of Ecology and Genetics, Uppsala University, Evolutionary Biology Centre, Norbyvägen 18D, 75105 Uppsala, Sweden

**Keywords:** Intralocus sexual conflict, Sexual antagonism, Same-sex sexual behavior, Pleiotropy, Mating strategy, Sexual selection, B-matrix, Genetic constraints, Artificial selection, Behavioral syndrome

## Abstract

**Background:**

Intralocus sexual conflict, arising from selection for different alleles at the same locus in males and females, imposes a constraint on sex-specific adaptation. Intralocus sexual conflict can be alleviated by the evolution of sex-limited genetic architectures and phenotypic expression, but pleiotropic constraints may hinder this process. Here, we explored putative intralocus sexual conflict and genetic (co)variance in a poorly understood behavior with near male-limited expression. Same-sex sexual behaviors (SSBs) generally do not conform to classic evolutionary models of adaptation but are common in male animals and have been hypothesized to result from perception errors and selection for high male mating rates. However, perspectives incorporating sex-specific selection on genes shared by males and females to explain the expression and evolution of SSBs have largely been neglected.

**Results:**

We performed two parallel sex-limited artificial selection experiments on SSB in male and female seed beetles, followed by sex-specific assays of locomotor activity and male sex recognition (two traits hypothesized to be functionally related to SSB) and adult reproductive success (allowing us to assess fitness consequences of genetic variance in SSB and its correlated components). Our experiments reveal both shared and sex-limited genetic variance for SSB. Strikingly, genetically correlated responses in locomotor activity and male sex-recognition were associated with sexually antagonistic fitness effects, but these effects differed qualitatively between male and female selection lines, implicating intralocus sexual conflict at both male- and female-specific genetic components underlying SSB.

**Conclusions:**

Our study provides experimental support for the hypothesis that widespread pleiotropy generates pervasive intralocus sexual conflict governing the expression of SSBs, suggesting that SSB in one sex can occur due to the expression of genes that carry benefits in the other sex.

**Electronic supplementary material:**

The online version of this article (doi:10.1186/s12862-016-0658-4) contains supplementary material, which is available to authorized users.

## Background

Selection for different alleles at the same locus in males and females can engender a genetic tug-of-war between the sexes, known as intralocus sexual conflict (IaSC) [1–6], where adaptations in one sex bear costs paid by the other. Such sexually antagonistic (SA) selection can maintain genetic variance in fitness at relatively high levels (e.g. [7–9]), suggesting that a sizeable part of the standing genetic variation in traits under selection will have opposing fitness effects in the two sexes. IaSC can be resolved by mechanisms allowing sex-specific expression of loci under SA selection, leading to the evolution of sex-limited genetic architecture and sexual dimorphism [10, 11]. Ultimate examples of such sex-differentiation are secondary sexual characters like male beetle horns and cervid antlers, or color ornamentation in male peacocks and guppies [12].

However, recent evidence suggests that pleiotropic constraints at SA loci may often hinder complete conflict resolution (e.g. [13–18]), and the evolution of sex-specific inheritance may shift conflict in favor of one sex rather than alleviate it [8, 11, 19]. Indeed, phenotypic traits with near sex-limited expression can still harbor significant amounts of SA genetic variation [20–22]. For example, artificial selection on the exaggerated male mandibles in the broad-horned flour beetle, *Gnatocerus cornutus*, has been shown to impose substantial fitness consequences in females, despite exaggerated mandibles only being expressed in males [23]. Thus, widespread pleiotropy may generate SA genetic variation in seemingly sex-limited traits via cross-trait intersexual genetic covariances, suggesting that an improved understanding of the evolution of these traits can be gained by adopting a multivariate approach taking fitness consequences in both sexes into account (e.g. [23–26]).

Here we used this conceptual framework to study the evolution of same-sex sexual behavior (SSB). We adopt the definition of SSB as behavior carried out towards individuals of the same sex that usually is part of the organism’s behavioral repertoire displayed in mating interactions with the opposite sex. Widespread in the animal kingdom, SSB has received much interest because it does not directly conform to classic models of selection and adaptation [27]. Reflecting its wide taxonomic range, a plethora of hypotheses have been proposed to explain the occurrence of SSB. However, while hypotheses invoking social dominance and kin selection could explain these behaviors in group-living animals that exhibit within-group competition and/or cooperative breeding, they carry little general explanatory power when applied to other taxa [27]. Despite this, SSB is particularly common in non-social animals, exemplified by the fact that same-sex mounting make up nearly 50 % of all mounting attempts in many insects [28].

More generally, SSB in the form of mounting behavior in male animals is often hypothesized to be a byproduct of perception errors resulting from selection for high male mating rates in polygamous species (the mistaken identity hypothesis: [28–31]). According to this hypothesis, male SSB evolves if the fitness cost associated with making errors is small relative to the costs of (i) missed heterosexual mating opportunities that stem from being selective and (ii) developing and maintaining cognitive abilities that allow perfect sex-discrimination. Interestingly, however, similar behavior has also been observed in female insects, despite mounting not being part of their behavioral repertoire in heterosexual interactions. As SSB usually occurs at much lower frequency in females and does not seem to have the same associated costs and benefits as for males (beetles: [32, 33]; flies: [32], wasps: [34]), other explanations have been invoked. In particular, it has been suggested that females in some taxa may benefit from mimicking males through SSB [35] or that low levels of female SSB may result from incomplete silencing of male-selected genes [36]. However, we know very little about the genetic architecture of SSB in females, and next to nothing about how selection acts on variation at underlying loci.

Models invoking SA selection, in which alleles encoding SSB in one sex have benefits when expressed in the other, show that SA selection on a shared genetic architecture in the sexes can act to maintain genetic polymorphisms underlying SSB [37], and thus implicate IaSC as an important element regulating the expression and evolution of SSB. Furthermore, SSB can be correlated to behavioral syndromes, which include not only courtship displays, but also activity rates, cognitive ability, and rate-dependent life history traits [27, 38]. Consequently, we hypothesized that the expression of SSB is, in addition to the more classic explanation invoking trade-offs and stabilizing selection exclusively in males (reviewed in: [28]), also governed by genes that experience SA selection through wide ranging pleiotropy encompassing other behavioral and physiological traits.

We tested our hypothesis in the seed beetle *Callosobruchus maculatus*, where females also display SSB at low frequencies when housed in same-sex groups [36]. This allowed us to apply sex-limited artificial selection on SSB (up-selection and down-selection) in males and females, in two parallel experiments. First, we tested whether SSB in males and females is governed by the same set of genes by comparing the response in SSB of the artificially selected focal sex to the correlated response in the unselected sex, in each of the two experiments. Second, guided by previous research in this [26, 36] and other systems [27–34], we focused on assessing sex-specific correlated responses to selection in two traits putatively functionally related to SSB and involved in IaSC in invertebrates; male perception (i.e. sex recognition) and locomotor activity. Third, we measured competitive lifetime reproductive success of males and females from all selection lines, allowing us to assess the prediction that variation in SSB should be linked to IaSC and SA fitness effects, partly mediated by the correlated responses in the two behavioral components. Specifically, we predicted that selection for increased male SSB would result in decreased male ability to discriminate between the sexes, leading to reduced male reproductive performance. We predicted the opposite effect on female reproductive performance, because female fecundity does not depend on sex recognition in this system, while increased cognitive ability has been repeatedly shown to be costly in female insects. We also predicted that selection for increased SSB in females would have negative effects on female reproductive performance if accompanied by a correlated increase in locomotor activity, because this trait has previously been shown to be negatively genetically correlated to female fecundity in *C. maculatus* [26]. At the same time, locomotion is an important component of male mate searching and scramble competition in this species, which would predict that males might instead benefit from increased locomotor activity.

We show that SSB in *C. maculatus* is governed by genes with shared phenotypic effects in the sexes, as well as by genes with sex-specific inheritance and/or expression. The two correlated traits, locomotor activity and male perception, both responded to artificial selection on SSB, but strikingly, showed fundamentally different relationships with fitness depending on whether artificial selection was applied to males or females. Despite these differences, however, the correlated responses had associated SA fitness effects in both male and female selection lines. Indeed, the sex selected for SSB tended to suffer a relative decrease in reproductive success whereas the opposite sex instead enjoyed an increase. These results provide experimental evidence suggesting that widespread pleiotropy generates IaSC at SSB loci via correlated behavioral traits, thereby supporting the hypothesis that IaSC can play an important role in the evolution of SSBs.

## Methods

### Study system

*Callosobruchus maculatus* (Coleoptera, Bruchinae) is a capital breeder, which lays most of its eggs during the first few days of life. Juvenile survival rates are usually well above 90 % (e.g. [39]), which makes them ideal for artificial selection experiments. This species is facultatively aphagous, i.e. adults do not require food or water to reproduce at high rates [40]. *C. maculatus* has an evolutionary history associated with human grain stores dating back several thousands of years (and tens of thousands of generations) [41], making them particularly suitable as a model system used in laboratory settings similar to these conditions (e.g. [42]).

Although female *C. maculatus* typically mate multiply, introducing post-copulatory sexual selection in males (e.g. [43, 44]), males will do so at much higher potential rates if given opportunity. Females are thus often seen resisting male mating attempts. Males often end up mounting other males in failed attempts to mate [36]. Males with entangled genitals, resulting from same-sex copulation attempts, are sometimes observed (personal observations). When females are housed in groups without harassing males, same-sex mounting is observed at low frequencies. In both sexes, a mounting beetle positions itself behind the rear end of the mounted beetle, standing on its hind legs and supporting itself by placing the front legs on the abdomen of the mounted beetle in a position that is indistinguishable from heterosexual mating. Male beetles often tap the mounted beetle with its front legs and/or antennae while females interrupt this behavior shortly after mounting another female and remain relatively motionless thereafter.

The study population was created by merging 41 isofemale lines that had been held at population sizes of 200–300 individuals for ca. 40 generations following their original establishment from a single natural population. The natural population was sampled from a small scale agricultural field close to Lomé, Togo (06°10′N 01°13′E), during October and November 2010 by I.A. Glitho in accordance with national legislation and permission from the local land owner, and sampled beetles were imported to Sweden under permission Dnr 30-4303/12 issued by the Swedish Board of Agriculture. We regenerated this population by randomly sampling ca. 20–40 individuals from each isofemale line and placing them together in a 1 L glass jar on 300 ml of *V. unguiculata* seeds, five generations prior to the artificial selection regime was implemented. The base population was thus held at ca 1000 beetles. The lines are thoroughly described in [45]. Given that they were sampled from the center of the species’ distribution, the created population on which we applied artificial selection presumably represents a genetically diverse natural population of *C. maculatus*. We note that because the lines originate from the very same natural population, we do not expect artificially elevated levels of epistatic variation in the base population.

### Pilot study

We performed a pilot study in the spring of 2013 in which we estimated the level of same-sex mounting and locomotor activity in males and females from each of the 41 isofemale lines. In total, 5752 beetles were assayed in groups of four, over three generations prior to implementing artificial selection. We identified partly sex-specific genetic variance and heritabilities for same-sex mounting, and observable levels of mounting in females, which motivated us to conduct sex-limited artificial selection on the behavior in both males and females. See Additional file 1: S1 for a full description of analyses and results.

### Artificial selection

We applied replicated artificial selection for the presence (up-selection) or absence (down-selection) of same-sex mounting behavior in males and females, over three consecutive generations (F1-F3). After the F3 generation we stopped the experiment as i) the selection procedure incorporating 16 replicate lines (see below) was very demanding, and ii) preliminary measures suggested very strong responses to artificial selection already after two generations (Additional file 1: Figure S2).

Beetles from the base population were split among four replicate 1 L glass jars in the (F0) generation prior to the artificial selection. From each of these replicates we created a selection line of each sex: treatment combination (male/female: up/down) in the F1 generation, resulting in a total of 16 artificial selection lines. Artificial selection was applied to three sets of beetles, each consisting of 24 individuals of the same sex, per replicate line. These 24 virgin naïve beetles were kept together in a 90 mm  Petri dish placed on a heating plate set at 29 °C (their typical rearing temperature: [45]). Beetles were free to move around and interact, ensuring that putatively correlated traits, such as sex recognition and general locomotor activity rates [27], could contribute to SSB (see below).

For up-selection, the four most frequent same-sex mounters out of the 24 beetles in a set were selected through sequential observation: In the initial bout, the first 16 (out of 24) beetles to mount another individual were transferred to a new 90 mm  Petri dish. In a second bout, the first 8 (out of 16) to mount were moved to a 30 mm  Petri dish (to keep densities approximately constant). Finally, the 4 (out of the remaining 8) beetles to first mount another beetle were selected to propagate the next generation. For down-selection, 16 mounters were removed until only eight non-mounters remained. Because at this point mounting was so uncommon among females, and we wanted to apply the same strength of selection and keep the same effective population size in male- and female-limited selection regimes, we randomly selected four out of the eight individuals from each of the three sets to form the new generation of down-selected beetles. Hence, from each of the 16 replicate lines, 12 out of 72 beetles were selected and paired with 12 unselected individuals of the opposite sex (in monogamous pairs) to form the new generation.

The number of propagated individuals was low in our selection lines (*N* = 24). To reduce the potential impact of genetic drift and inbreeding, in each generation of propagation, we made sure that each pair in each line contributed with an equal amount of potential recruits (six) to the next generation. In addition, we always crossed offspring of couples originating from the different three bouts in the previous generation of selection in a round-robin design, which precluded sib-mating. *C. maculatus* is resistant to inbreeding; noticeable depression requires several consecutive generations of full-sib mating in this (Grieshop et al., *submitted*) as well as other (e.g., [46]) populations. These considerations, in combination with the fact that lifetime reproductive success in our lines (see Results) was high relative to other observations in this population (e.g. [45]), suggest that inbreeding depression did not affect our results. We also note that a previous study successfully used this same protocol to apply sex-limited artificial selection on male longevity in another population of *C. maculatus* [47].

### Same-sex mounting and locomotor activity

After two generations of relaxed selection (i.e. in the F5), during which all lines were kept in larger 1 L glass jars at population sizes of around 300 beetles, we assayed same-sex mounting and locomotor activity of both sexes. Four virgin individuals of the same sex were put in a 30 mm  Petri dish placed on a heating plate set at 29 °C. After 5 min of acclimation, the total number of mountings and movements were registered continuously over a period of 10 min for each dish; i.e. the response was noted as number of movements/mountings for all four beetles combined per the 10 min of observation. For each of the 16 lines, six dishes (four beetles in each) were observed per sex, totaling 192 dishes.

### Male perception

We assayed male (same-sex) mounting of other males in the presence of available females in the F6 offspring. We created arenas by gluing a dead virgin male and a dead virgin female reference beetle to the bottom surface of a 90 mm Petri dish at equidistance to its margins. These decoys were killed by flash freezing in liquid nitrogen to preserve their chemical composition, which may be important in sex-discrimination in this species [48]. After flash freezing, the beetles were stored over night at −20 °C before being glued to the arena just prior to the experiments. Arenas were replaced after three hours of assays to make sure fresh beetles were used as decoys. In total we used 24 arenas.

Two males from a given selection line were simultaneously introduced into an arena at equidistance from the glued male and female and then observed for 10 min during which their combined total number of mountings performed on the glued male and female, respectively, were recorded. We used two males in each trial since single beetles can stay inactive for long periods of time. Indeed, we discarded many (foremost down-selected) male pairs where there was no male activity (nor mounting); in total we analyzed 129 out of a total of 208 observed assays (see Additional file 1: Figure S6 for same-sex and opposite-sex mounting rates calculated including all 208 assays).

To avoid bias, all behaviors (same-sex mounting, locomotor activity and male perception) were scored by a naïve observer (having no knowledge of selection line identity).

### Lifetime reproductive success

In the generation following termination of artificial selection (i.e. in F4), we assayed adult lifetime reproductive success (LRS) in competitive settings, in males and females separately. Virgin focal individuals originating from the 16 replicate lines were competed against beetles from a reference population in 90 mm  Petri dishes with ad libitum (ca. 100) black eyed beans as egg-laying substrate. We used the unselected base population as the competitive background.

In the female assays, one adult focal female (i.e. from one of the selection lines) was placed together with a reference female, that had been sterilized, and two reference males. We sterilized female reference beetles (and male reference beetles in the male assays; see below) with a 100Gy dose of gamma radiation using a cesium-137 source. This dose has been shown to leave both sexes of this population sterile for their lifetime without noticeable effects on longevity (I. Martinossi and D. Berger, unpublished data). All focal beetles were newly emerged (less than 24 h old), whereas individuals from the base population were 0–48 h old, and all beetles were virgin and kept individually until introduced in the assays. This setting allowed the focal female to compete with the sterilized reference female over matings, as male ejaculates can have positive effects on female fecundity in this species (e.g. [49–51]). However, multiple mating can also have negative effects on females (e.g. [52]) because male genitalia are harmful [53] and possibly because the ejaculate may contain harmful compounds [54], and hence such effects were also part of the design. To assay male LRS, one focal male was placed together with a sterilized reference male competitor and two fertile virgin reference females. Male LRS was measured as the total number of offspring sired by the focal male, produced by the two females. Sterilized reference males’ sperm is motile and able to fertilize eggs, but the zygotes die; thus, this integrative protocol captures both pre- and post-copulatory sexual selection (e.g. [45, 55]). In both male and female assays, all individuals were left together until their natural death.

After all offspring had emerged from the fitness assays, they were frozen at −20 °C for subsequent offspring counts. We aimed at determining LRS of two males and two females from each of the 12 F4 full-sib families from each of the 16 lines. Eleven observations were discarded due to fungus growth on beans, resulting in a total of 757 assays, evenly split across the two sexes and 16 selection lines.

### Statistics

Responses to sex-limited artificial selection in same-sex mounting, locomotor activity, and LRS were analyzed in partially nested ANOVAs using expected mean squares estimation for balanced data, implemented in R v. 3.2.3 [56]. We included “sex selected” (artificial selection applied on either male or female mounting), “sex assayed” (trait measured in either males or females), “treatment” (artificial selection up/down on mounting) and their interactions, as factors. Line identity, nested in “sex selected” and “treatment”, and crossed by “sex assayed”, were incorporated as the random effects used to evaluate significance. Male trait values were much higher than female values for same-sex mounting and locomotor activity. These variables were therefore log-transformed (i.e., we compared proportional changes in the traits across sexes and selection regimes), which also rendered the residuals of our models approximately normal. To model LRS, we searched for the most appropriate transformation to achieve normality using Box-Cox power transformation available in the MASS package for R [57], giving: (Offspring + 5)^1.5. Significance was evaluated using partial F-tests with the denominator degrees of freedom based on the number of replicate lines. Because nested ANOVAs in R were developed for strictly balanced data, and our data on LRS were missing 11 out of 768 observations, we also replicated this analysis using SYSTAT [58] that performs nested ANOVAs using REML estimation while handling unbalanced designs. However, these two approaches gave identical results (Additional file 1: S6).

Male perception (proportion of female mountings) was analyzed in generalized linear mixed effects models using a binomial error distribution and a logit-link function, implemented in the lme4 package [59] in R. Model specification was identical to that for the other three traits except that “sex assayed” was not included as the trait was only measured in males, and that the date and time of each trial was included as a fixed factor and covariate respectively, and arena identity was included as an additional random effect. Significance of fixed effects was evaluated using likelihood ratio tests based on REML and type II Wald chi-square statistics using the car package [60]. To complement this analysis and control for over-dispersion in the data, we also performed generalized linear models, leaving out random effects (which approached the parameter boundary: see Results), allowing a quasi-binomial error distribution for the response. Significance of fixed effects was evaluated using F-ratio tests.

Finally, we estimated sex-specific genetic covariance between the four assayed traits based on responses to artificial selection by correlating trait means across the 16 selection lines for each sex. To identify SA selection, we put special emphasis on testing for sex-differences in the sign of the slope of the regression of LRS on each of the three behavioral traits, which would signify IaSC. In addition, we also looked for differences in this regression depending on which sex that had been selected for SSB. Thus, these analyses of covariance (ANCOVAs) always included LRS as the response variable and one of the three behavioral traits as covariate, and “sex selected”, “sex assayed”, and their interactions, as factors. Because male perception only was measured in males, line identity was included as a random effect crossed by sex to account for the paired male and female observations from each line when analyzing this trait.

## Results

### Sex-specific responses to selection: same-sex mounting

**Fig 1:**
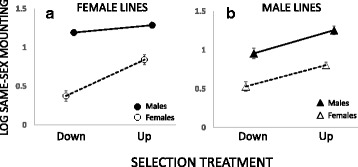
Response to sex-limited artificial selection on female (**a**) or male (**b**) same-sex mounting, assayed in females (*open symbols*) and males (*closed symbols*). Plotted is the mean for each selection treatment: sex combination ± 1SE based on line means on a log10 scale

Irrespective of the artificial selection treatment, same-sex mounting was much more pronounced in males than in females (*F*_1,12_ = 171.7, *p* < 0.001). Up-selection resulted in substantially higher mounting rate relative to down-selection (*F*_1,12_ = 64.0, *p* < 0.001). However, this response was stronger in the sex upon which selection had been applied, especially so for assayed females (sex selected*sex assayed*treatment: *F*_1,12_ = 5.57, *p* = 0.036), suggesting both shared and sex-limited genetic variation for the trait (Fig. 1, full summary of statistics in Additional file 1: S3).

### Sex-specific correlated responses: locomotor activity

**Fig 2:**
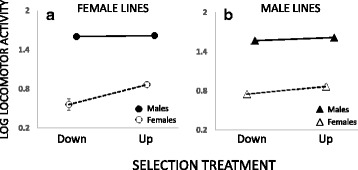
Correlated responses in locomotor activity of females (*open symbols*) and males (*closed symbols*) from female (**a**) and male (**b**) selection lines. Plotted is the mean for each selection treatment-sex combination ± 1SE based on line means on a log10 scale

Irrespective of the artificial selection treatment, locomotor activity was much higher in males than in females (*F*_1, 12_ = 891.8, *p* < 0.001). Selection for increased same-sex mounting resulted in increased locomotor activity relative to down-selection (*F*_1, 12_ = 19.83, *p* < 0.001). However, this correlated response was mainly seen in females (sex assayed*treatment: F_1, 12_ = 8.51, p = 0.013). (Fig. 2, full summary of statistics in Additional file 1: S4).

### Sex-specific correlated responses: male perception

**Fig. 3:**
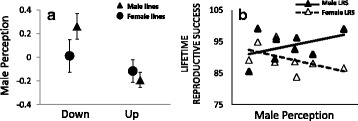
In **a** Correlated response of male perception (female/total number of mountings), represented by model residuals. Plotted is the mean for each selection treatment ± 1SE based on means for the four replicate lines. Male perception responded to selection on males, with up-selection leading to a decrease in perception (i.e. increased perception error), but showed little response to selection on females. In **b** scores for male perception are plotted against scores for male (*filled triangles*) and female (*open triangles*) lifetime reproductive success for the male selection lines

The ratio of female to total number of mountings in the two-choice arena test had decreased in response to up-selection relative to down-selection for same-sex mounting (Chi-2 = 7.17, df = 1, *p* = 0.007). This decrease was due to overall higher rates of indiscriminant mounting with respect to sex in up-selected males (Additional file 1: S5). Although there was no significant difference in the strength of the correlated response between the two experiments (sex selected *treatment: Chi-2 = 2.46, df = 1, *p* = 0.12), separate analyses showed that male perception had responded readily to selection on males (Chi-2 = 10.1, df = 1, *p* = 0.0015), but not females (Chi-2 = 0.36, df = 1, *p* = 0.55) (Fig. 3a, Additional file 1: Table S5). There was a tendency for overdispersion of model residuals. As the variance between selection lines nested within selected sex was low (effect of line: Chi-2 = 0.40, df = 2, *P* = 0.82), model likelihoods were likely evaluated against the residual variance rather than against line variance, making it possible that overdispersion may have affected our analyses. To check the robustness of our results, we therefore applied generalized linear models (removing random effects), allowing quasi-binomial error distributions correcting for overdispersion. Reassuringly, these models gave the same qualitative results (male experiment: *F*_1, 68_ = 5.08, *p* = 0.027; female experiment: *F*_1, 53_ = 1.88, *p* = 0.18).

### Sex-specific correlated responses: lifetime reproductive success

**Fig 4:**
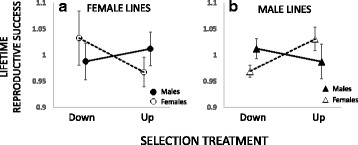
Correlated responses in relative lifetime reproductive success (LRS) of females (*open symbols*) and males (*closed symbols*) in female (**a**) and male lines (**b**). Plotted is the mean for each selection treatment-sex combination ± 1SE based on line means

In both the male and female experiment, the observed correlated responses in LRS in one sex tended to show the opposite responses in the other (Fig. 4), in line with ongoing IaSC over genes regulating same sex mounting. Indeed, there was evidence for IaSC in terms of a statistically significant interaction between “assayed sex” and “treatment” in the male experiment (*F*_1, 6_ = 6.91, *p* = 0.039). This effect was, however, not statistically significant when selection was applied to females (*F*_1, 6_ = 2.69, *p* = 0.15). Most strikingly, the sex-specific responses in LRS after having applied selection on males were in the opposite direction relative to the responses observed when selection was applied on females (selected sex*assayed sex*treatment: *F*_1, 12_ = 7.88, *p* = 0.016). Such a pattern suggests that different sets of genes, with different sex-specific fitness effects, responded to selection in the male and female experiment (Fig. 4, Additional file 1: S6). While these results indicate SA fitness effects of variation in same-sex mounting, the effects of treatment (selection up or down on same-sex mounting) in each sex and selection experiment considered separately were relatively weak and seen only for female LRS (Effect of selection treatment: females from male selection lines: *F*_1, 6_ = 5.83, *p* = 0.052; females from female selection lines: *F*_1, 6_ = 1.26, *p* = 0.305; males from male selection lines: *F*_1, 6_ = 0.48, *p* = 0.516; males from female selection lines: *F*_1, 6_ = 0.39, *p* = 0.556). Thus, the strongest effect on LRS from applying artificial selection on same-sex mounting, a trait foremost expressed in males under natural conditions, was seen in females originating from male selection lines.

### Sex-specific genetic architectures and sexually antagonistic selection

**Fig. 5:**
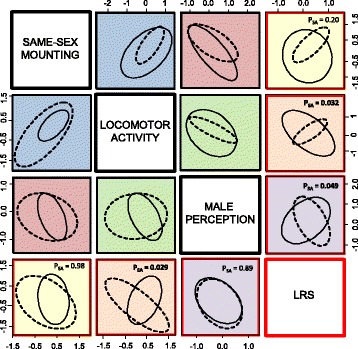
Genetic covariances between the assayed traits based on selection line means in males (*full ellipses*) and females (*hatched ellipses*). Confidence ellipses are fitted to data from male selection lines plotted above, and female selection lines plotted below, the diagonal. Corresponding trait covariances are matched for color to ease comparisons of sex-specific covariances across male and female selection lines. There was more genetic (co)variance in SSB and the correlated traits for males when sex-limited artificial selection was applied on males (above diagonal: full ellipses > hatched ellipses), and for females when selection was applied on females (below diagonal: hatched ellipses > full ellipses), implying that sex-limited genes underlie SSB in *C. maculatus*. Panels containing covariances between the three behavioral traits and LRS inform about selection on the traits and are highlighted by red framing. The respective *P*-value for a sex:trait interaction in linear regressions of LRS on each trait, which if significant signifies sexually antagonistic selection, is given in each panel (see text for more details). Notably, both locomotor activity (*orange panels*), and male perception (*purple panel above diagonal*), show SA genetic covariance with LRS, suggesting ongoing IaSC over genes encoding SSB in *C. maculatus*. Note that male perception was scored only in males and is correlated across sexes for the female data below the diagonal. Because male trait values were an order of magnitude greater than female trait values for locomotor activity and same-sex mounting, all traits were mean centered and variance standardized for each sex separately before plotting

To gain further insights into the genetic architecture of, and selection on same-sex mounting and its underlying components, we continued to explore the correlated evolutionary responses by estimating sex-specific genetic covariance between the three behavioral traits and LRS, based on means for each of the 16 selected replicate lines and two sexes (Fig. 5).

Genetic covariance between same-sex mounting and LRS was only weak and non-significant in itself (Fig. 5, Additional file 1: Table S7a). More strikingly however, a three-way interaction between sex selected, sex assayed and locomotor activity explained the majority of genetic variance in LRS between selection lines (*F*_1, 24_ = 11.7, *p* = 0.0022, Additional file 1: Table S7b). Artificial selection on same-sex mounting had generated SA genetic covariance between LRS and locomotor activity among both male selected (sex assayed*locomotor activity: *F*_1, 12_ = 5.92, *p* = 0.032) and female selected lines (sex assayed*locomotor activity: *F*_1, 12_ = 6.12, *p* = 0.029), in each case signified by the slope of the regression of LRS on locomotor activity having opposite signs in the two sexes (Fig. 5)*.* However, for both sexes, the relationship between LRS and locomotor activity was reversed between male and female selection lines (hence the significant three-way interaction), again indicating that very different genes encoding locomotor activity had been selected in the two experiments (Fig. 5, Additional file 1: Table S7b). Male perception showed no genetic covariance with LRS overall (Additional file 1: Table S7c). However, when analyzing male and female lines separately, there was evidence that artificial selection for same-sex mounting had generated SA genetic covariance between male perception and LRS in male lines (sex assayed*male perception: *F*_1, 6_ = 6.04, *p* = 0.049; again signified by the slope of the regression of LRS on male perception having opposite signs in the two sexes: Figs. 3b and 5). This was not the case in female lines (sex assayed*male perception: *F*_1, 6_ = 0.02, *p* = 0.89), consistent with the correlated response of male perception being limited to male lines (see Fig. 3a). These last results thus demonstrate that artificial selection on same-sex mounting generated SA genetic fitness variation via correlated responses in the underlying behavioral components locomotor activity and male perception.

## Discussion

Multivariate genetic constraints can set fundamental limits to adaptive evolution [61] and several recent studies have highlighted the importance of taking a multivariate approach to study constraints on sex-specific adaptation via cross-trait intersexual genetic covariances (i.e. the B-matrix; [10]). Here we used this conceptual framework to increase our limited understanding of the evolutionary basis of same-sex sexual behavior, a trait that is predominantly expressed in males and widespread among animal taxa. Traditionally, SSB has been mostly studied in males, with the leading explanation for it being “perception error” (i.e. imperfect sex recognition) coupled with strong selection for high male mating rate and the trade-off between costs of occasional same-sex mounting versus missed opportunity costs [29]. Poor sex recognition can certainly affect rates of male-male mounting and studies in *Drosophila* provide an emerging understanding of the proximate basis for male SSB via this link (reviewed in: [27, 28]). However, recent studies have only begun to uncover the underlying genetic basis of SSB in males [36, 62] and the evolution of female SSB remains largely unexplored. Moreover, most previous studies have stopped short of exploring the link between standing genetic variation in SSB and fitness.

Theoretical work suggests that SSB in both sexes can evolve when alleles that increase fitness in one sex result in SSB in the other [37]. However, there is little empirical evidence supporting the notion that genetic variation in SSB is related to male or female fitness [63] (but see: [62]). In this study, we provided a comprehensive three-step attempt at elucidating the evolution of both male and female SSB in the seed beetle *C. maculatus*. First, we quantified sex-specific genetic variation and heritability for SSB across 41 isofemale lines derived from a natural population. Second, we used this population to conduct separate artificial selection experiments on SSB in each sex, complemented by sex-specific fitness assays. Third, we assayed correlated genetic responses to artificial selection in the putatively functionally associated traits – male perception/sex recognition and locomotor activity. Overall, our results suggest that SSB is encoded by alleles with both shared and sex-limited effects. While fitness effects associated directly with genetic responses in SSB were weak (especially so in males: Fig. 4), strong responses in the correlated traits resulted in SA fitness effects, apparently through distinct sets of genes when selection was applied in males versus females (Fig. 5). This is consistent with the hypothesis that SA selection on multiple underlying behavioral/physiological components is responsible for maintaining a significant fraction of the observed standing genetic variation in SSB in this natural population.

Up-selection on male SSB reduced male sex recognition whereas females from the same lines enjoyed a relative increase in LRS. Cognitive performance should be costly [64] and recent work on *D. melanogaster* fruit flies have demonstrated that males evolving without sexual selection show reduced cognitive performance during courtship and are less able to direct their mating efforts towards receptive females [65]. Our results are broadly in line with these findings and further suggest that the expression of genes either in tight linkage disequilibrium or directly involved in male sex recognition can be costly if expressed in females (Fig. 3b), but the proximate basis underlying this result remains to be explored. For now, the cost of sex recognition remains a hypothesis and it is possible that increased male SSB results in increased female fecundity in beetles via different pleiotropic effects on female physiology and/or life-history.

Contrary to male-limited selection, up-selection on female SSB did not impair male sex recognition and instead reduced female LRS relative to down-selected lines. The reduced female LRS was coupled with a prominent increase in female locomotor activity, which is in line with previous results demonstrating SA genetic variation and female detriment associated with high activity levels in other laboratory populations of *C. maculatus* [26] and *D. melanogaster* [66]. In contrast, female locomotor activity did not respond as readily to male-limited artificial selection on SSB, and the genetic correlation between female locomotor activity and LRS was instead positive across male selected lines. The different correlated responses of locomotor activity in the male and female experiment is striking but consistent with the highly sex-specific genetic architecture and rich number of sex-specific quantitative trait loci (QTLs) for locomotor activity reported in *Drosophila* [67], and highly sex-specific genetic responses in locomotor activity observed in previous artificial selection experiments on another population of *C. maculatus* [26]. Thus, taken together, the results from our two selection experiments suggest that female and male same-sex mounting are at least partly encoded by different sets of genes, each with SA fitness effects.

Indeed, the implication of abundant SA genetic variation is in line with theoretical expectations for SSB [37, 63] and more generally for traits under SA selection [1, 6, 11] (but see: [8]). The conditions under which SA selection can maintain polygenic variation for SSB are more restrictive than for the single locus case modeled by Gavrilets and Rice [37] (compare: [7] and [68]). However, these conditions widen, also for the multilocus case, when there is sex-specific dominance for fitness [8, 9]. Nevertheless, our demonstration of SA selection on genes underlying SSB does not necessarily imply that SA selection alone is responsible for the maintenance of standing genetic variation in SSB. For example, genotype-environment interactions [45] or negative frequency-dependent selection [69] may contribute substantially to genetic polymorphism at SA loci. Indeed, SA selection on genetic variation underlying alternative male mating strategies has now been identified in several study systems (e.g. soay sheep: [70]; horned beetles: [71]; bulb mites: [72]; and salmon: [73]) and it seems likely that also within-sex antagonistic pleiotropy as well as frequency-dependent selection contribute to maintaining these polymorphisms. Male mating phenotypes are typically related to behavioral and life history syndromes [3], as also demonstrated in *C. maculatus* [26, 47, 74]. These composite phenotypes often encompass traits such as locomotor activity, metabolism and reproductive rate, as well as aggressiveness and dominance behaviors [3, 38], that also are predicted to affect SSB [27, 28], providing a general mechanistic link between the expression of SSB and the evolution of male mating strategies.

We observed strong responses to artificial selection after only three generations. Correlated responses to short term artificial selection may sometimes be poor predictors of long term responses [75, 76] and evolutionary constraints [77–79] (but see: [61]), because weak physical linkage and genetic (co)variances can both easily be altered by persistent selection [80]. This is an issue that is likely to pertain in its most severe form to experiments using single-generation breeding designs to study genetic covariance matrices (i.e. the G-matrix) [75, 76]. Our experiment aligns itself with a large number of studies using short-term artificial selection, and like the results from these studies, our findings thus need to be interpreted with some caution. However, the fact that we here deliberately targeted traits that i) previously have been identified as hotspots for IaSC [26, 66], and ii) are predicted to be functionally related to SSB and, thus, are a priori expected to be regulated by a shared set of pleiotropically acting genes [27], would suggest that the reported covariances are tell-tale signs of non-transient genetic constraints [76]. We also note that the observed responses in same-sex mounting and locomotor activity were well predicted from our estimates of sex-specific genetic (co)variances based on the isofemale line analysis (Additional file 1: S1).

## Conclusions

Consistent with our findings, Hoskins et al. [62] recently found an influence of sex-linked genetic variance in male SSB on female fecundity using four inbred lines from the *Drosophila* Genetic Reference Panel. However, the fact that male reproductive success was not scored made it difficult to assess the relative importance of overdominance versus SA selection in maintaining genetic variance in SSB [62]. Our data suggest that both shared and sex-limited SSB alleles have strong pleiotropic effects and that sex-limited selection on this behavioral trait can have SA fitness effects via cross-trait intersexual genetic correlations. This implication is consistent with other recent studies highlighting the important role of wide-ranging pleiotropy in generating IaSC (e.g. [23–26]), and thus, serves more generally as an example of the broad impact of IaSC on the evolution of sexually selected traits. While few studies have provided information on SA effects and associated responses in correlated phenotypes coupled to SSB as done here, our findings in many ways parallel one of the emerging explanations for the evolutionary maintenance of homosexuality in humans [63], pointing to a general influence of IaSC on the expression of different forms of SSB across diverse animal taxa.

### Ethics

Collection and experiments on beetles were performed in accordance with local national legislations.

### Consent to publish

N.A.

### Availability of data and material

The datasets supporting the conclusions of this article are included within the article and its additional files; Additional file 2.

## Additional files

Additional file 1: S1. Summary of Pilot study – Estimating broad sense heritability and sex-specific genetic variation in same-sex mounting and locomotor activity using isofemale lines. **S2:** Figure on preliminary responses in same-sex mounting (Mean ± 1SE) to sex-limited artificial selection over the first two (out of three) generations. **S3:** Full summary statistics and figure on responses to selection in same-sex mounting. **S4:** Full summary statistics and figure on responses to selection in locomotor activity. **S5:** Full summary statistics and figure on responses to selection in male perception. **S6:** Full summary statistics and figure on responses to selection in lifetime reproductive success. **S7:** Full summary statistics on sex-specific genetic covariances between behavioral traits and LRS. (DOCX 355 kb) Additional file 2: Data: Locomotor activity & Same-Sex Mounting, Lifetime Reproductive Success, Male Perception Error. (ZIP 6 kb)

## Abbreviations

IaSC: intralocus sexual conflict
LRS: (competitive) lifetime reproductive success
SA: sexual antagonism/sexually antagonistic (fitness effects)
SSB: same-sex sexual behavior
