# Supplementary material for: Sexually antagonistic selection on genetic variation underlying both male and female same-sex sexual behavior
Source: BMC Evol Biol. 2016 May 13;16:88. doi: 10.1186/s12862-016-0658-4 (PMC4866275; doi:10.1186/s12862-016-0658-4)
Supplement: Additional file 1: S1. — Summary of Pilot study – Estimating broad sense heritability and sex-specific genetic variation in same-sex mounting and locomotor activity using isofemale lines. S2: Figure on preliminary responses in same-sex mounting (Mean ± 1SE) to sex-limited artificial selection over the first two (out of three) generations. S3: Full summary statistics and figure on responses to selection in same-sex mounting. S4: Full summary statistics and figure on responses to selection in locomotor activity. S5: Full summary statistics and figure on responses to selection in male perception. S6: Full summary statistics and figure on responses to selection in lifetime reproductive success. S7: Full summary statistics on sex-specific genetic covariances between behavioral traits and LRS. (DOCX 355 kb) [file 12862_2016_658_MOESM1_ESM.docx]

**Additional file 1:** *Sexually antagonistic selection on genetic variation underlying both male and female same-sex sexual behavior*

**S1:** Pilot study – Estimating broad sense heritability and sex-specific genetic variation using isofemale lines.

Same-sex mounting behavior and locomotor activity was quantified in the 41 isofemale lines by censoring 5752 beetles, kept in groups of four, over three consecutive generations (blocks) prior to the main sex-limited artificial selection experiments. The traits were assessed in the same manner as described for the selected populations. This data indicated a moderate intersexual genetic correlation (rMF) for SSB, and also preliminary indications of sex-differences in the magnitude of genetic variation for SSB, suggesting both shared and sex-specific genetic architectures underlying male and female SSB.

We used the lme4 package (Bates et al. 2011) for the statistical computing freeware R (R Core Team 2013) to construct linear mixed effect models using isofemale line crossed by sex to partition variance in SSB and locomotor activity among its genetic (among isofemale lines) and environmental (within isofemale line) components. In addition to these effects of main interest, we also controlled for the placement of the petri-dishes and sex-specific effects of experimental block (generation), waiting (acclimation) time of the beetles prior to censoring, and the time of day and date of the trials. We performed two complementary analyses for SSB. First we estimated sex-specific genetic variance in SSB using the total counts of same-sex mountings. Secondly we estimated variance in SSB whilst adding locomotor activity as a covariate in the model, hence estimating occurrence of SSB controlling for differences mediated by movement. However, these two analyses gave similar outcome, indicating that SSB is not solely driven by variation in locomotor activity.

To test if the intersexual genetic correlation was significantly positive, we compared the likelihood of models estimating the correlation to models fixing it at zero. Similarly, to test for sex-specific genetic variance, we compared models estimating genetic variance in each sex separately to models estimating it across both sexes simultaneously. These analyses are summarized briefly below.

Same-sex mounting controlled for locomotor activity:

modMount3a <- glmer(mount ~ LOGmove*sex + place*block + sex*LOGwait + block*LOGwait + sex*shift + (0+sex|line) + (0+sex|date) + (0+as.numeric(sex=="f")|ID) + (0+as.numeric(sex=="m")|ID), family="poisson", data= maraOUT)

AIC BIC logLik deviance

3822 3975 -1882 3764

Random effects:

Groups Name Variance Std.Dev. rMF

ID as.numeric(sex == "m") 7.3361e-01 8.5651e-01

ID as.numeric(sex == "f") 8.6703e-01 9.3114e-01

**line sexf 1.7771e-01 4.2156e-01**

**sexm 9.0999e-02 3.0166e-01 0.397**

date sexf 1.6698e-10 1.2922e-05

sexm 5.7871e-02 2.4056e-01 NA

Number of obs: 1438, groups: ID, 1438; line, 41; date, 12

Fixed effects:

Estimate Std. Error z value Pr(>|z|)

(Intercept) 1.52274 0.13845 10.999 < 2e-16 ***

LOGmove 0.80303 0.06250 12.849 < 2e-16 ***

sex[T.m] -0.59699 0.14988 -3.983 6.8e-05 ***

place[T.2] 0.23093 0.12681 1.821 0.06860 .

place[T.3] 0.22521 0.12592 1.788 0.07370 .

place[T.4] 0.30888 0.12580 2.455 0.01408 *

block[T.three] -0.46991 0.16571 -2.836 0.00457 **

block[T.two] -0.22163 0.15024 -1.475 0.14018

LOGwait -0.09349 0.06664 -1.403 0.16062

shift[T.pm] -0.10948 0.09093 -1.204 0.22859

LOGmove:sex[T.m] 0.06222 0.07947 0.783 0.43366

place[T.2]:block[T.three] -0.33080 0.21627 -1.530 0.12612

place[T.3]:block[T.three] -0.09992 0.21046 -0.475 0.63495

place[T.4]:block[T.three] -0.08069 0.21066 -0.383 0.70171

place[T.2]:block[T.two] -0.36948 0.19141 -1.930 0.05358 .

place[T.3]:block[T.two] -0.31513 0.19120 -1.648 0.09931 .

place[T.4]:block[T.two] -0.18182 0.18973 -0.958 0.33792

sex[T.m]:LOGwait 0.15667 0.07526 2.082 0.03736 *

block[T.three]:LOGwait -0.16805 0.09674 -1.737 0.08236 .

block[T.two]:LOGwait -0.21747 0.08617 -2.524 0.01161 *

sex[T.m]:shift[T.pm] -0.05427 0.12124 -0.448 0.65442

Same-sex mounting not controlled for locomotor activity:

modMount4a <- glmer(mount ~ place*block + sex*LOGwait + block*LOGwait + sex*shift + (0+sex|line) + (0+sex|date) + (0+as.numeric(sex=="f")|ID) + (0+as.numeric(sex=="m")|ID), family="poisson", data = maraOUT)

AIC BIC logLik deviance

4258 4401 -2102 4204

Random effects:

Groups Name Variance Std.Dev. rMF

ID as.numeric(sex == "m") 1.1759096 1.084394

ID as.numeric(sex == "f") 1.2046529 1.097567

**line sexf 0.2859648 0.534757**

**sexm 0.1267444 0.356012 0.357**

date sexf 0.0047765 0.069112

sexm 0.0366673 0.191487

Number of obs: 1438, groups: ID, 1438; line, 41; date, 12

Fixed effects:

Estimate Std. Error z value Pr(>|z|)

(Intercept) 0.83350 0.15434 5.400 6.65e-08 ***

place[T.2] 0.29615 0.14939 1.982 0.047439 *

place[T.3] 0.39150 0.14837 2.639 0.008323 **

place[T.4] 0.41573 0.14825 2.804 0.005043 **

block[T.three] -0.72673 0.18542 -3.919 8.88e-05 ***

block[T.two] -0.51071 0.16822 -3.036 0.002398 **

sex[T.m] 0.94522 0.14892 6.347 2.19e-10 ***

LOGwait -0.28019 0.07531 -3.720 0.000199 ***

shift[T.pm] 0.03467 0.10227 0.339 0.734573

place[T.2]:block[T.three] -0.40747 0.24970 -1.632 0.102709

place[T.3]:block[T.three] -0.10519 0.24383 -0.431 0.666186

place[T.4]:block[T.three] -0.10032 0.24400 -0.411 0.680973

place[T.2]:block[T.two] -0.33788 0.22354 -1.512 0.130661

place[T.3]:block[T.two] -0.37633 0.22357 -1.683 0.092317 .

place[T.4]:block[T.two] -0.22223 0.22180 -1.002 0.316366

sex[T.m]:LOGwait 0.25377 0.08679 2.924 0.003455 **

block[T.three]:LOGwait -0.13739 0.11236 -1.223 0.221426

block[T.two]:LOGwait -0.14650 0.10036 -1.460 0.144360

sex[T.m]:shift[T.pm] -0.26865 0.13971 -1.923 0.054487 .

Locomotor activity:

modMount5a <- glmer(move ~ place*block + sex*LOGwait + block*LOGwait + sex*shift +

(0+sex|line) + (0+sex|date)+ (0+as.numeric(sex=="f")|ID) + (0+as.numeric(sex=="m")|ID) ,family="poisson", data = maraOUT)

AIC BIC logLik deviance

3800 3942 -1873 3746

Random effects:

Groups Name Variance Std.Dev. rMF

ID as.numeric(sex == "m") 0.5845594 0.764565

ID as.numeric(sex == "f") 1.3623190 1.167184

**line sexf 0.1074195 0.327749**

**sexm 0.1153063 0.339568 0.752**

date sexf 0.0000000 0.000000

sexm 0.0059681 0.077253

Number of obs: 1438, groups: ID, 1438; line, 41; date, 12

Fixed effects:

Estimate Std. Error z value Pr(>|z|)

(Intercept) -0.36126 0.13697 -2.638 0.008351 **

place[T.2] 0.02709 0.12705 0.213 0.831164

place[T.3] 0.18363 0.12510 1.468 0.142151

place[T.4] 0.11412 0.12582 0.907 0.364418

block[T.three] -0.41906 0.15077 -2.779 0.005445 **

block[T.two] -0.47036 0.13794 -3.410 0.000650 ***

sex[T.m] 2.83881 0.11375 24.958 < 2e-16 ***

LOGwait -0.42462 0.08160 -5.204 1.95e-07 ***

shift[T.pm] 0.35949 0.12366 2.907 0.003649 **

place[T.2]:block[T.three] 0.02777 0.20371 0.136 0.891576

place[T.3]:block[T.three] 0.13533 0.19983 0.677 0.498254

place[T.4]:block[T.three] 0.09765 0.20104 0.486 0.627175

place[T.2]:block[T.two] 0.08765 0.18553 0.472 0.636616

place[T.3]:block[T.two] -0.07702 0.18474 -0.417 0.676744

place[T.4]:block[T.two] -0.04933 0.18672 -0.264 0.791622

sex[T.m]:LOGwait 0.32788 0.08489 3.863 0.000112 ***

block[T.three]:LOGwait 0.04554 0.09083 0.501 0.616133

block[T.two]:LOGwait 0.09000 0.08592 1.047 0.294890

sex[T.m]:shift[T.pm] -0.49338 0.13933 -3.541 0.000398 ***

**Testing for positive rMFs:**

Same-sex mounting controlled for locomotor activity:

modMount3a <- glmer(mount ~ LOGmove*sex + place*block + sex*LOGwait + block*LOGwait + sex*shift + (0+sex|line) + (0+sex|date) + (0+as.numeric(sex=="f")|ID) + (0+as.numeric(sex=="m")|ID), family="poisson", data = maraOUT)

modMount3c <- glmer(mount ~ LOGmove*sex + place*block + sex*LOGwait + block*LOGwait + sex*shift + (0+as.numeric(sex=="f")|line) + (0+as.numeric(sex=="m")|line) + (0+sex|date) + (0+as.numeric(sex=="f")|ID) + (0+as.numeric(sex=="m")|ID), family="poisson", data = maraOUT)

anova(modMount3a, modMount3c)

Df AIC BIC logLik Chisq Pr(>Chisq)

modMount3c 28 3822.7 3970.3 -1883.3

modMount3a 29 3821.8 3974.7 -1881.9 2.8508 0.09133 .

Same-sex mounting not controlled for locomotor activity:

modMount4a <- glmer(mount ~ place*block + sex*LOGwait + block*LOGwait + sex*shift + (0+sex|line) + (0+sex|date) + (0+as.numeric(sex=="f")|ID) + (0+as.numeric(sex=="m")|ID),family="poisson", data = maraOUT)

modMount4c <- glmer(mount ~ place*block + sex*LOGwait + block*LOGwait + sex*shift +

(0+as.numeric(sex=="f")|line) + (0+as.numeric(sex=="m")|line) + (0+sex|date) + (0+as.numeric(sex=="f")|ID) + (0+as.numeric(sex=="m")|ID),family="poisson", data = maraOUT)

anova(modMount4a, modMount4c)

Df AIC BIC logLik Chisq Pr(>Chisq)

modMount4c 26 4258.5 4395.6 -2103.3

modMount4a 27 4258.2 4400.5 -2102.1 2.3359 0.1264

Locomotor activity:

modMount5a <- glmer(move ~ place*block + sex*LOGwait + block*LOGwait + sex*shift + (0+sex|line) + (0+sex|date)+ (0+as.numeric(sex=="f")|ID) + (0+as.numeric(sex=="m")|ID) ,family="poisson", data = maraOUT)

modMount5c <- glmer(move ~ place*block + sex*LOGwait + block*LOGwait + sex*shift +

(0+as.numeric(sex=="f")|line) + (0+as.numeric(sex=="m")|line) + (0+sex|date)+ (0+as.numeric(sex=="f")|ID) + (0+as.numeric(sex=="m")|ID) ,family="poisson", data = maraOUT)

anova(modMount5a, modMount5c)

Df AIC BIC logLik Chisq Pr(>Chisq)

modMount5c 26 3805.9 3943.0 -1877

modMount5a 27 3800.1 3942.4 -1873 7.8217 0.005162 **

**Testing for sex-specific genetic variances:**

Same-sex mounting controlled for locomotor activity:

modMount3b <- glmer(mount ~ LOGmove*sex + place*block + sex*LOGwait + block*LOGwait + sex*shift +

(1|line) + (0+sex|date) + (0+as.numeric(sex=="f")|ID) + (0+as.numeric(sex=="m")|ID), family="poisson", data = maraOUT)

modMount3c <- glmer(mount ~ LOGmove*sex + place*block + sex*LOGwait + block*LOGwait + sex*shift + (0+as.numeric(sex=="f")|line) + (0+as.numeric(sex=="m")|line) + (0+sex|date) + (0+as.numeric(sex=="f")|ID) + (0+as.numeric(sex=="m")|ID), family="poisson", data = maraOUT)

anova(modMount3b, modMount3c)

Df AIC BIC logLik Chisq Pr(>Chisq)

modMount3b 27 3833.1 3975.4 -1889.5

modMount3c 28 3822.7 3970.3 -1883.3 12.386 0.0004326 ***

Same-sex mounting not controlled for locomotor activity:

modMount4b <- glmer(mount ~ place*block + sex*LOGwait + block*LOGwait + sex*shift +

(1|line) + (0+sex|date) + (0+as.numeric(sex=="f")|ID) + (0+as.numeric(sex=="m")|ID),family="poisson", data = maraOUT)

modMount4c <- glmer(mount ~ place*block + sex*LOGwait + block*LOGwait + sex*shift +

(0+as.numeric(sex=="f")|line) + (0+as.numeric(sex=="m")|line) + (0+sex|date) + (0+as.numeric(sex=="f")|ID) + (0+as.numeric(sex=="m")|ID),family="poisson", data = maraOUT)

anova(modMount4b, modMount4c)

Df AIC BIC logLik Chisq Pr(>Chisq)

modMount4b 25 4276.8 4408.6 -2113.4

modMount4c 26 4258.5 4395.6 -2103.3 20.31 6.585e-06 ***

Locomotor activity:

modMount5b <- glmer(move ~ place*block + sex*LOGwait + block*LOGwait + sex*shift +

(1|line) + (0+sex|date)+ (0+as.numeric(sex=="f")|ID) + (0+as.numeric(sex=="m")|ID) ,family="poisson", data = maraOUT)

modMount5c <- glmer(move ~ place*block + sex*LOGwait + block*LOGwait + sex*shift +

(0+as.numeric(sex=="f")|line) + (0+as.numeric(sex=="m")|line) + (0+sex|date)+ (0+as.numeric(sex=="f")|ID) + (0+as.numeric(sex=="m")|ID) ,family="poisson", data = maraOUT)

anova(modMount5b, modMount5c)

Df AIC BIC logLik Chisq Pr(>Chisq)

modMount5b 25 3798.0 3929.8 -1874

modMount5c 26 3805.9 3943.0 -1877 0 1

**S2:** Preliminary responses in same-sex mounting (Mean ± 1SE) to sex-limited artificial selection over the first two (out of three) generations. Responses were quantified instantaneously by measuring the time it took to pick out the first 8 same-sex mounting individuals during the selection trials; note the reversed scale on the Y-axis. The responses were relativized to the response in the first (unselected) generation, for male and female selection separately.

**S3:** Full summary statistics of analysis on same-sex mounting.

Partially Nested ANOVA; Table of effects.

Error: pop

Df Sum Sq Mean Sq F value Pr(>F)

treatment 1 15.918 15.918 63.974 3.77e-06 ***

sex_sel 1 0.763 0.763 3.067 0.105

treatment:sel_sel 1 0.013 0.013 0.053 0.822

Residuals 12 2.986 0.249

Error: pop.:sex_ass

Df Sum Sq Mean Sq F value Pr(>F)

sex_ass 1 57.18 57.18 171.650 1.81e-08 ***

sex_ass:treatment 1 0.80 0.80 2.399 0.147

sex_ass:sex_sel 1 1.45 1.45 4.355 0.059 .

sex_ass:treatment:sex_sel 1 1.85 1.85 5.568 0.036 *

Residuals 12 4.00 0.33

Error: Within

Df Sum Sq Mean Sq F value Pr(>F)

Residuals 160 38.52 0.2407

**Fig S3:** Female (a) and male (b) responses to sex-limited artificial selection (male selection lines = black symbols, female selection lines = white symbols). Plotted are the means of each of the 16 selection lines ± 1SE. Each sex responded to selection on the other sex. However, the responses were stronger in the sex upon which the artificial selection was applied, signifying both shared and private genetic variation for same-sex mounting in the two sexes.


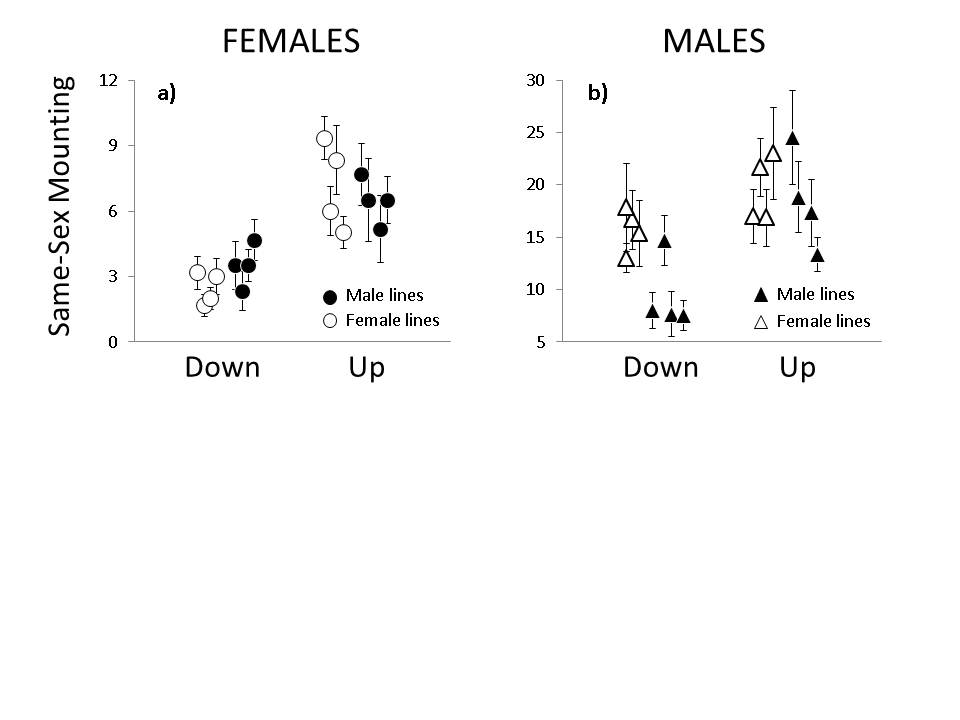


**S4:** Full summary statistics of analysis on locomotor activity.

Partially Nested ANOVA; Table of effects.

Error: pop

Df Sum Sq Mean Sq F value Pr(>F)

treatment 1 3.183 3.183 19.833 0.0008 ***

sex_sel 1 0.200 0.200 1.246 0.286

treatment:sex_sel 1 0.236 0.236 1.471 0.249

Residuals 12 1.926 0.160

Error: pop:sex_ass

Df Sum Sq Mean Sq F value Pr(>F)

sex_ass 1 173.86 173.86 891.793 1.24e-12 ***

sex_ass:treatment 1 1.66 1.66 8.509 0.0129 *

sex_ass:sex_sel 1 0.49 0.49 2.530 0.138

sex_ass:treatment:sex_sel 1 0.49 0.49 2.500 0.140

Residuals 12 2.34 0.19

Error: Within

Df Sum Sq Mean Sq F value Pr(>F)

Residuals 160 26.39 0.165

**Fig S4:** Locomotor activity. Female (a) and male (b) correlated responses to sex-limited artificial selection (male selection lines = black symbols, female selection lines = white symbols) on same-sex mounting. Plotted are the means of each of the 16 selection lines ± 1SE. Females responded to selection on males, but not vice versa, and generally, female locomotor activity showed stronger correlated responses to selection on same-sex mounting than did male locomotor activity.


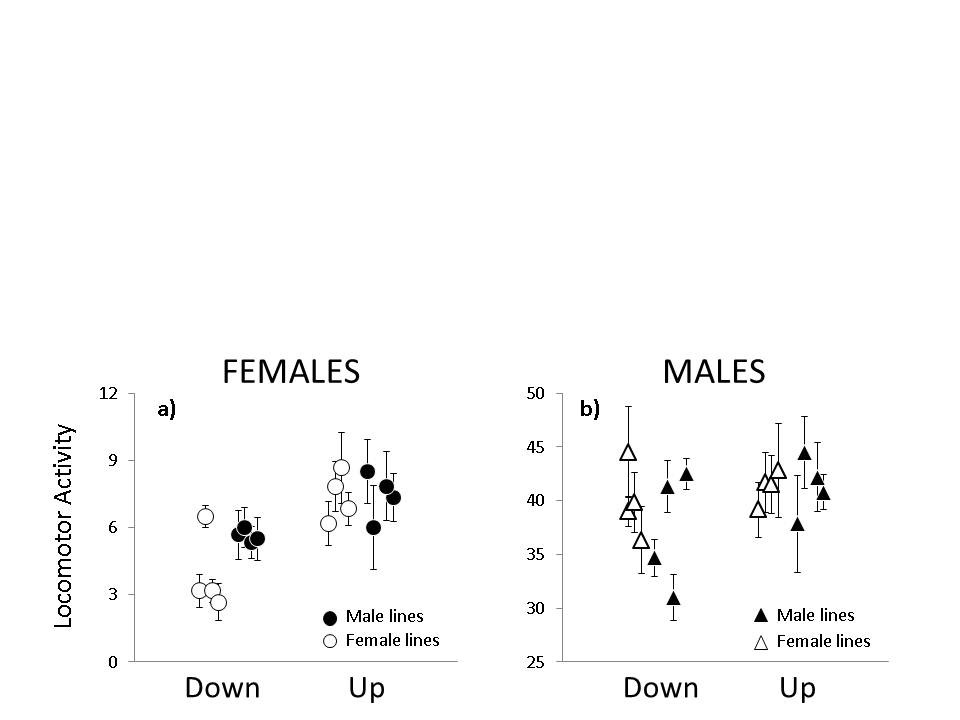


**S5:** Full summary statistics of analysis on male perception.

Analysis of Deviance Table (Type II Wald chi-square tests)

**All data:**

Chisq Df P

sex selected 1.0871 1 0.297

treatment 7.1663 1 0.0074 **

time 0.5153 1 0.473

date 0.9116 1 0.340

sex selected: treatment 2.4613 1 0.117

**Male lines:**

Chisq Df P

treatment 10.13 1 0.0015 **

date 6.580 1 0.0103 *

time 4.928 1 0.0264 *

**Female lines:**

Chisq Df P

treatment 0.363 1 0.547

date 0.556 1 0.456

time 2.083 1 0.149


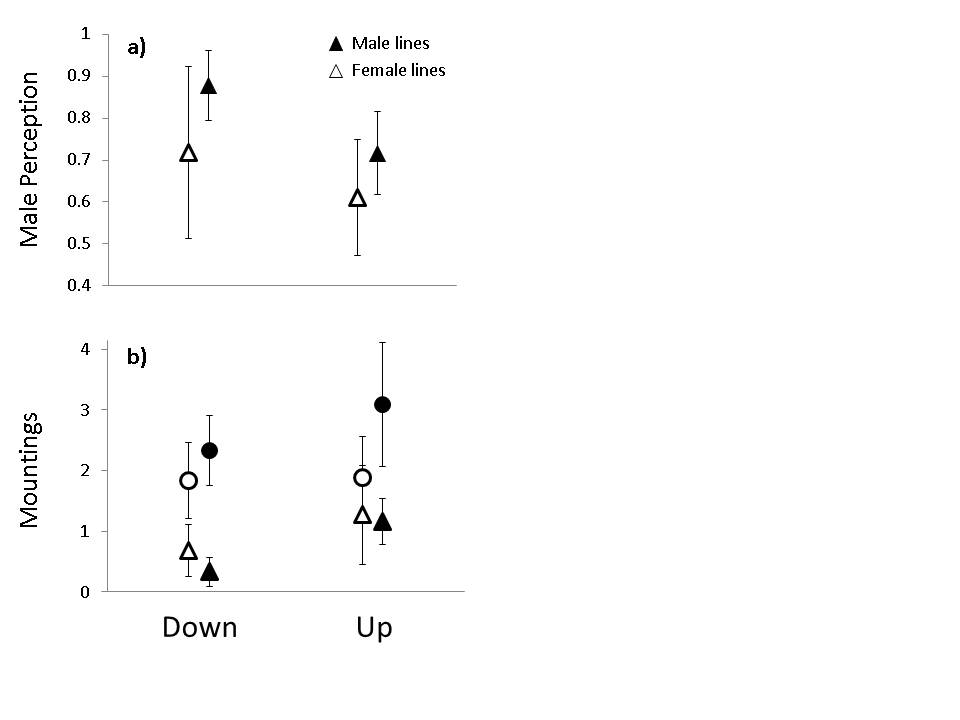


**Fig. S5:** Male perception (defined as the ratio of female to total number of mountings). Correlated responses in males to sex-limited artificial selection (male selection lines = black symbols, female selection lines = white symbols) on same-sex mounting. Plotted are raw means of the four selection treatments ± 95% confidence limits. a) Male perception responded readily to selection on males, with up-selection leading to a decrease in perception (i.e. increased perception error) relative to down-selection. Male perception showed little response to selection on females. b) Mounting of females (circles) were more common than same-sex mountings of males (triangles), but only male-limited up-selection tended to increase mounting of females, whereas both male- and female up-selection increased same-sex mounting. The results from male-limited selection (black symbols) illustrate a positive relationship between mountings of females and erroneous same-sex mountings of males, and thus conform to “the perception error”-hypothesis.

**S6:** Full summary statistics of analysis on lifetime reproductive success (LRS).

Partially Nested ANOVA; Table of effects. [for balanced data in R]:

Error: pop

Df Sum Sq Mean Sq F value Pr(>F)

treatment 1 1159 1159 0.006 0.941

sex_sel 1 403432 403432 2.033 0.188

treatment:sex_sel 1 75242 75242 0.379 0.553

Residuals 12 1785921 198436

Error: pop:sex_ass

Df Sum Sq Mean Sq F value Pr(>F)

sex_ass 1 1600331 1600331 21.93 0.0005 ***

treatment:sex_ass 1 400 400 0.005 0.942

sex_sel:sex_ass 1 149489 149489 2.049 0.178

treatment:sex_sel:sex_ass 1 574838 574838 7.879 0.0158 *

Residuals 12 875535 72961

Error: Within

Df Sum Sq Mean Sq F value Pr(>F)

Residuals 725 1.04e+08 143497

Partially Nested ANOVA; REML TYPE III SS, Table of effects. [for unbalanced data in SYSTAT]

df den.df F-Ratio P-Value

sex_sel 1 12 2.031 0.180

treatment 1 12 0.002 0.969

sex_ass 1 12 22.06 0.001

treatment*sex_sel 1 12 0.366 0.556

sex_sel:sex_ass 1 12 2.195 0.164

treatment:sex_ass 1 12 0 0.995

treatment:sex_sel:sex_ass 1 12 7.865 0.016

**Fig S6:** Female (a) and male (b) correlated responses to sex-limited artificial selection (male selection lines = black symbols, female selection lines = white symbols) on same-sex mounting behavior. Plotted are the means of each of the 16 selection lines ± 1SE. The sexes showed highly sexually antagonistic responses in LRS. When comparing females from male lines, up-selection increased female LRS relative to down-selection. However, the opposite was true when comparing females from female selection lines (a). In males, responses in LRS were weak (b). Nevertheless, the documented responses were always in opposite direction to that observed in the corresponding comparison for females (compare a and b). Thus, there seems to be two sets of genes with sex-specific inheritance governing same-sex mounting behavior, both with sexually antagonistic effects on fitness.


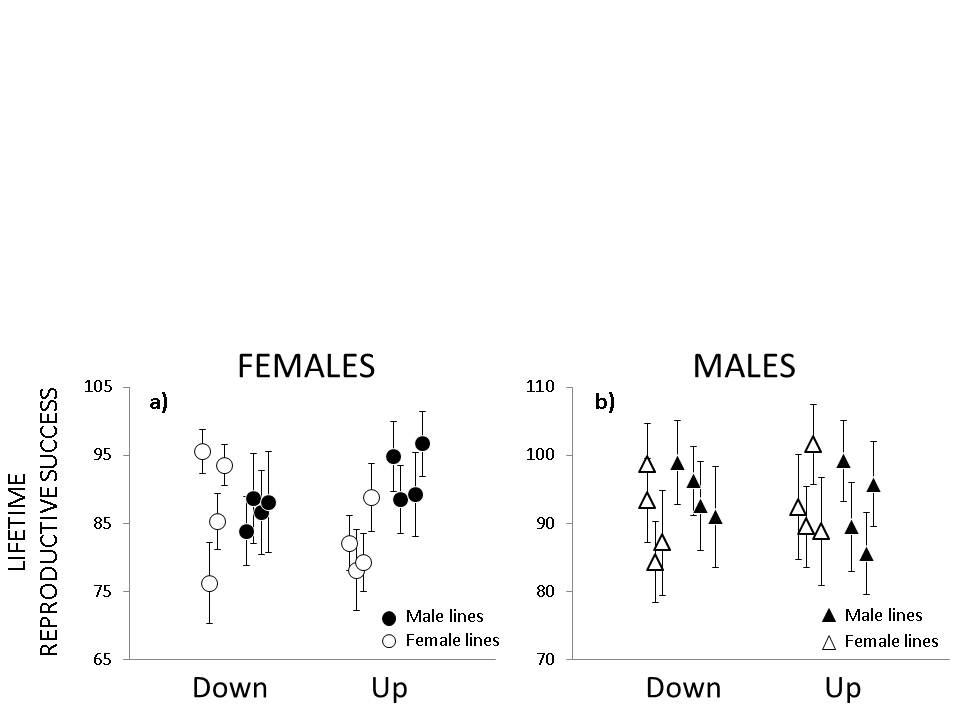


**S7:** Full summary statistics of analysis on sex-specific genetic covariances between behavioral traits and LRS

Table S7a: same-sex mounting:

ANCOVA Table:

**All data:**

Df Mean Sq F value Pr(>F)

sex_ass 1 0.000 0.000 1.00000

sex_sel 1 2.229 2.188 0.152

mounting 1 0.011 0.011 0.918

sex_ass:sex_sel 1 0.472 0.463 0.503

sex_ass:mounting 1 0.005 0.004 0.948

sex_sel:mounting 1 2.306 2.226 0.145

sex_ass:sex_sel:mounting 1 0.531 0.521 0.477

Residuals 24 1.019

**Male Lines:**

Df Mean Sq F value Pr(>F)

sex_ass 1 0.212 0.337 0.572

mounting 1 0.519 0.826 0.381

sex_ass:mounting 1 1.183 1.885 0.195

Residuals 12 0.627

**Female Lines:**

Df Mean Sq F value Pr(>F)

sex_ass 1 0.212 0.150 0.705

mounting 1 1.200 0.851 0.374

sex_ass:mounting 1 0.001 0.000 0.983

Residuals 12 1.410

Table S7b: Locomotor Activity:

ANCOVA Table:

**All data:**

Df Mean Sq F value Pr(>F)

sex_ass 1 0.000 0.000 1.000

sex_sel 1 2.229 3.177 0.087 .

activity 1 1.157 1.649 0.211

sex_ass:sex_sel 1 0.815 1.162 0.292

sex_ass:activity 1 0.665 0.948 0.340

sex_sel:activity 1 0.085 0.121 0.731

sex_ass:sex_sel:activity 1 8.213 11.708 0.00224 **

Residuals 24 0.702

**Male Lines**:

Df Mean Sq F value Pr(>F)

sex_ass 1 0.212 0.417 0.531

activity 1 0.143 0.281 0.606

sex_ass:activity 1 3.000 5.915 0.0316 *

Residuals 12 0.507

**Female Lines:**

Df Mean Sq F value Pr(>F)

sex_ass 1 0.211 0.236 0.636

activity 1 1.884 2.104 0.173

sex_ass:activity 1 5.485 6.123 0.0292 *

Residuals 12 0.896

Table S7c: Male Perception:

ANCOVA Table:

**All data:**

Error: pop

Df Mean Sq F value Pr(>F)

sex_sel 1 2.229 1.736 0.212

perception 1 0.591 0.460 0.510

sex_sel:perception 1 1.118 0.871 0.369

Residuals 12 1.284

Error: pop:sex_ass

Df Mean Sq F value Pr(>F)

sex_ass 1 0.000 0.000 1.000

sex_ass:sex_sel 1 0.423 0.612 0.449

sex_ass:perception 1 0.992 1.435 0.254

sex_ass:sex_sel:perception 1 0.944 1.366 0.265

Residuals 12 0.691

**Male Lines:**

Error: pop

Df Mean Sq F value Pr(>F)

perception 1 0.003 0.003 0.958

Residuals 6 0.902

Error: pop:sex_ass

Df Mean Sq F value Pr(>F)

sex_ass 1 0.212 0.667 0.4451

sex_ass:perception 1 1.915 6.043 0.0492 *

Residuals 6 0.317

**Female Lines:**

Error: pop

Df Mean Sq F value Pr(>F)

perception 1 1.706 1.024 0.351

Residuals 6 1.666

Error: pop:sex

Df Mean Sq F value Pr(>F)

sex_ass 1 0.212 0.198 0.672

sex_ass:perception 1 0.021 0.020 0.892

Residuals 6 1.066
